# Comparative analysis of toxicity reduction of wastewater in twelve industrial park wastewater treatment plants based on battery of toxicity assays

**DOI:** 10.1038/s41598-019-40154-z

**Published:** 2019-03-06

**Authors:** Yue Yu, Bing Wu, Linmiao Jiang, Xu-Xiang Zhang, Hong-Qiang Ren, Mei Li

**Affiliations:** State Key Laboratory of Pollution Control and Resource Reuse, School of the Environment, Nanjing University, Nanjing, 210023 P.R. China

## Abstract

Wastewater treatment plants (WWTPs) in industrial parks provide centralized treatment for industrial and domestic wastewater. However, the information on toxicity reduction of wastewater and its correlation with treatment process in industrial park is limited. This study compared the toxicity reduction of wastewater in 12 industrial park WWTPs based on battery of toxicity assays. Nine toxic endpoints involving microorganism, phytoplankton, zooplankton, plant and human cell lines were applied. All the influents of WWTPs induced high toxicities, which were significantly reduced after the treatments from 7 of the studied WWTPs. However, the effluents of five WWTPs induced higher toxicity in one or more toxic endpoints compared to the influents. This study also found that most of anaerobic-anoxic-oxic (A^2^/O)-based processes had good removal efficiency of wastewater toxicity, while the sequencing batch reactor (SBR)-based processes had the lowest removal efficiency. Moreover, low correlation coefficients were obtained among all toxic endpoints, indicating that battery of toxicity assays was necessary to completely characterize the toxicity and risk of wastewater in industrial parks. This study shed new lights to the toxicity reduction of wastewater and its correlation with treatment process, which is very useful for the design, management and operation of WWTPs in industrial parks.

## Introduction

Industrial park is the main production form in China. Nowadays, more than 20000 industrial parks have been approved by the government. These industrial parks are making great contribution to China’s economy. In 2011, 54 national economic and technological development zones occupied less than 0.5% of the urban land but contributed to 8.8% of China’s Gross Domestic Product^1^. The zoned pattern of industrial park accompanies centralized treatment of industrial wastewater pooled from various factories located in the industrial park. The 2012 Report on the State of the Environment in China showed that 80% of 22.16 billion tons of industrial wastewater flew into the centralized wastewater treatment plants^2^. Thus, more concerns should be raised on the fate of wastewater from industrial parks.

Wastewater treatment plants (WWTPs) in industrial parks provide centralized treatment for a variety of industrial wastewater as well as domestic wastewater. In the case of China, effluents of WWTPs are discharged when reaching the *Discharge Standard of Pollutants for Municipal Waste Water Treatment Plant of China*, where 12 fundamental water quality parameters and several heavy metals are controlled. However, industrial wastewater usually contains a complex composition of pollutants, such as persistent organic pollutants and endocrine disrupting chemicals, which have high human and ecological risks. Many literatures have proven that some of these pollutants can not be completely eliminated by the WWTPs^3–5^. Their removal efficiency is relative with the treatment technologies used in the WWTPs^6,7^. At present, many sewage treatment processes are used in industrial park WWTPs in China, including anaerobic-anoxic-oxic (A^2^/O), sequencing batch reactor (SBR), and oxidation ditch^8^. However, current studies on removal efficiency focus on chemical oxygen demand (COD), nitrogen, phosphorous, which could not indicate the reduction of toxicity and environmental risk of wastewater.

Chemical analyses can only measure limited pollutants, which are not always in accordance with toxic effects of wastewater^9^. Toxicity tests provide adverse biological effects on the mixed pollutants in WWTPs, which can be used to indirectly indicate the removal efficiency of wastewater^10,11^. A range of toxicity tests have been developed to evaluate the adverse effects of wastewater based on the use of bacteria, microalgae, invertebrate, plant and fish in earlier studies, while some of them focus on only one or a limited set of test species^12,13^. Species respond differently to chemicals, therefore it is necessary to conduct multiple toxicity tests with a variety of species. Over the past twenty years, a battery of bioassays have been applied in the assessment of toxicity of wastewater treatment^14–16^. These bioassays could effectively provide toxicity evaluation of WWTPs effluents.

This study chose 12 centralized WWTPs in industrial parks located in Jiangsu province, China to compare the toxicity of influents and effluents based on a battery of bioassays. Nine acute and genetic toxicity assays involving in microorganism, phytoplankton, zooplankton, plant and human cell lines were applied. Correlations among toxic endpoints were analyzed. The influents and effluents in the 12 industrial WWTPs were compared to explore the factors, which can affect the toxicity level of effluents. Although every industrial park includes many types of factories, all WWTPs studied in this research have the large bulking tank to mix wastewater from different factories, which ensures the stability of mixed wastewater entered into wastewater treatment process. So, the results of this study could indicate the real condition of toxicity reduction in different WWTPs, and provide useful information for management of WWTPs in industrial parks.

## Materials and Methods

### Sample collection and preparation

**Figure 1:**
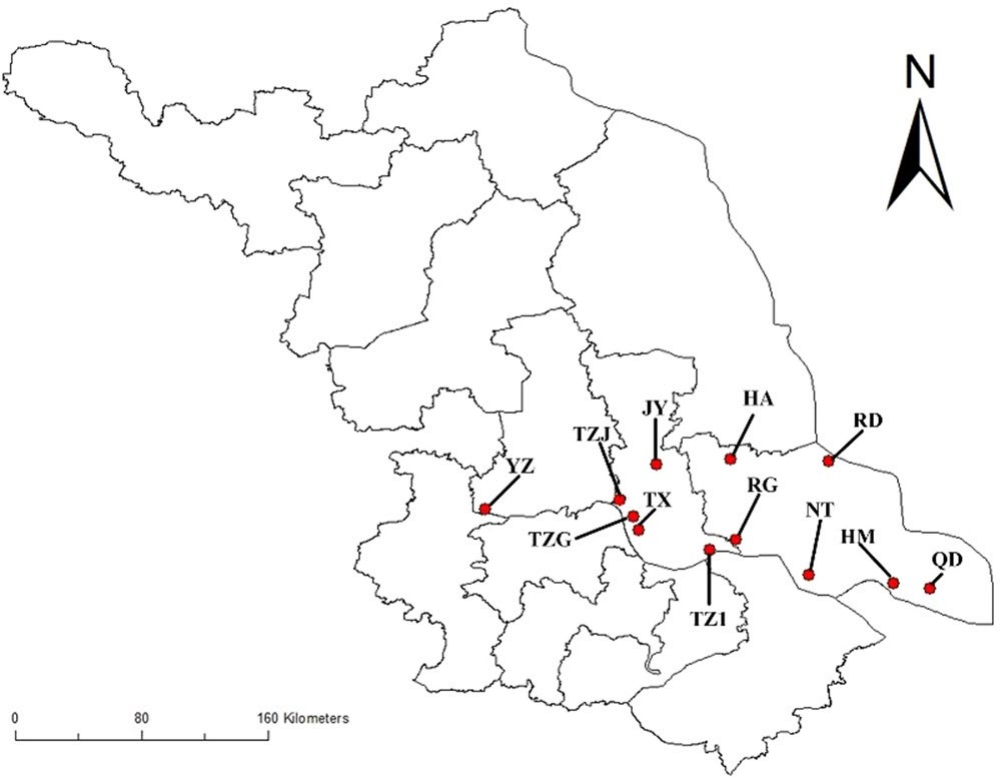
Sampling location of 12 WWTPs studied in this study. All the WWTPs located in Jiangsu Province, China.

Influent and effluent samples in the 12 industrial park WWTPs were collected on October 2015. These WWTPs locate in the low reach of Yangtze River, which is one of the most economically developed regions in China. Basic information on these WWTPs is shown in Fig. 1 and Table S1. A total of 20 L wastewater samples were collected in each WWTP and transported to laboratory under 4 °C. The pH values and alkalinity in the influents and effluents are shown in the Table S2. The COD, total nitrogen (TN) and total phosphorus (TP) values in effluents ranged from 8.84–87.78 mg/L, 3.48–25.62 mg/L and 0.06–0.63 mg/L, respectively (Table S3). All the samples were filtered by 0.45 μm membrane filter. Then the filtered wastewater samples were directly used to perform toxicity assays, except for the cytotoxicity assays.

### Bioluminescence inhibition assay

Bioluminescence inhibition assays were carried out using the luminescent bacteria *Photobacterium phosphoreum T3* spp. The luminescent bacteria were obtained from the Nanjing Institute of Soil Science, Chinese Academy of Sciences (Nanjing, China). The test was performed as described in previous study^15^. The inhibition percentage (%I) was determined by comparing the response given by a saline control solution to that corresponding to a sample, which is defined as: I(%) = [1-(sample light/control light)] × 100. Each sample was tested in quintuplicate.

### Acute toxicity assays

The *Euglena gracilis*, *Tetrahymena Thermophila* and *Daphnia magna straus* were applied to determine acute toxicity of the wastewater samples. The *E*. *gracilis*, *T*. *Thermophila* were obtained from the Freshwater Algae Culture Collection at the Institute of Hydrobiology, Chinese Academy of Sciences (Wuhan, China). Their acute toxicities were indicated by their growth inhibition^15,17^. The absorbance was measured by a microplate reader (Synergy H1, BioTek, USA) at 610 nm and 492 nm for *E*. *gracilis* and *T*. *Thermiphila*, respectively. The wastewater samples without cells were measured to eliminate the color effects of samples. The growth inhibition rate was expressed by I% = (A_x_ − A_xo_)/(A_c_ − A_co_) × 100%, where I is the inhibition rate, A_x_ is the absorbance of cells cultured in sample x, A_xo_ is the absorbance of water sample x without cells, A_c_ is the absorbance of cells cultured in culture medium, and A_co_ is the absorbance of the culture medium without cells.

Acute toxicity on *D*. *magna* was indicated by immobilization inhibition test. Ten mL of water sample and 5 active individuals of *D*. *magna* (12 h after birth) were added into 6-well plates with three replicates. Aerated tap water was used as the control. After 24 h and 48 h of exposure (12 h illumination/d), the number of inhibited individuals in each group was recorded. Inhibition was characterized by immobilization with no response to the movement of water^18^. The acute toxicity was expressed by inhibition rate, I% = (number of inhibited individuals / number of tested individuals) × 100%.

### Genotoxicity assays

Micronucleus test for *Vicia faba* was performed based on the reported method^19,20^. Randomly selected views on the slides were monitored to determine the number of micronucleated cells. Total number of scored cells was taken from 6 separated seedlings for each group and 1000 cells from each treated root tip were observed. Genotoxicity was expressed by the rate of micronucleus, MCN*‰* = (number of cells with micronucleus/total number of scored cells) × 1000*‰*. Pollution index (PI) was calculated by ratio of the mean MCN*‰* between the treatment and control groups.

Alkaline single-cell gel electrophoresis (comet assay) of *E*. *gracilis* was performed following method described in previous study with some modifications^21^. Slides were examined with a fluorescent microscope (BX41, Olympus, Japan). Three slides for each treatment were prepared and at least 50 cells were analyzed for each slide. Images were analyzed by using the Comet Assay Software Project (CASP). The tail moment (TM) and olive tail moment (OTM) were used to evaluate the genotoxicity of water samples.

### Cytotoxicity assays

All the wastewater samples filtered by 0.45 μm membrane were extracted by solid-phase extraction based on our previous study^22^. In brief, samples (5 L) were adjusted to pH = 2 and loaded onto the C_18_ extraction column (1 g, 3 mL) at a flow rate of 3 mL/min. After dried under vacuum the cartridges were eluted with 20 mL hexane, 20 mL hexane: methylene chloride (4:1, V: V), 10 mL methylene chloride and 20 mL methanol: methylene chloride (4:1, V: V) under gravity, respectively. The eluates were evaporated under a gentle stream of nitrogen and reconstituted into 1 mL of 2% dimethyl sulfoxide (DMSO), then stored under −20 °C until usages.

Human hepatoma cell line HepG2 was chosen to indicate the cytotoxicity of water samples. The HepG2 cells were purchased from KeyGEN Biotech (Nanjing, China), and maintained in Dulbecco’s modified Eagles medium (DMEM) containing 10% fetal bovine serum (FBS) at 37 °C and 5% CO_2_. The HepG2 cells were cultured for 24 h in the 96-well microplate (10000 cells/well) and then exposed to concentrated water sample for another 24 h. The cell viability was determined by cell counting kit-8 (CCK-8, Dojindo Molecular Technologies, Inc. Japan). The cells treated by CCK-8 treatment were measured at 450 nm with a microplate reader (Synergy H1, BioTek, USA) and cell viability of treated groups was shown as the relative absorbance compared to control group.

Intracellular ROS level was measured by cell dye 2,7-dichloro-fluorescin diacetate (DCFDA, Life Technologies, USA). To reduce the errors from loss of cell number during sample exposures, DNA fluorescent dye Hoechst 33342 (YeaSen Biotechnology, China) was used to normalize the DCF fluorescence value by counting the number of HepG2 cells in 96-plate wells^23^. Fluorescence values of DCF and Hoechst 33342 were measured using a microplate reader (Synergy H1, BioTek, USA). The excitation/emission wavelengths for DCF and Hoechst 33342 were 485/530 nm and 350/460 nm, respectively.

Mitochondrial membrane potential, an early indicator of cell apoptosis, was measured by a JC1 detection kit (KeyGEN, Nanjing). Two fluorescence values of JC1 were measured by a microplate reader (Synergy H1, BioTek). The excitation and emission wavelengths for green fluorescence of JC1 were 485 and 530 nm, while those for red fluorescence were 530 and 590 nm, respectively. Ratio of red to green fluorescence values was used to indicate the mitochondrial membrane potential.

The ATP-binding cassette (ABC) transporters was chosen as a target to analyze the adverse effect of water samples on transmembrane proteins by measuring accumulation of cell probe Calcein-AM (CAM, Dojindo Molecular Technologies, Japan)^24,25^. The Hoechst 33342 was also applied to normalize CAM fluorescence values. The excitation/emission wavelengths of CAM were 485/530 nm. Further, chemosensitive effects of ABC transporter inhibition was determined by using arsenic (As) as target chemical. The conjunction of As and glutathione (GSH) is a known substrate of ABC transporters^26^. Arsenic oxide was obtained from NSI Solution Inc. (Raleigh, USA) The As at nontoxic concentration (1 μmol/L) and wastewater samples were exposed to HepG2 cells simultaneously, after 24 h exposure, intracellular ROS level was chosen as the toxic endpoint.

### Statistical Analysis

For all assays (except bioluminescence inhibition test), three independent trials were performed, and each trial was replicated six times. All the data were expressed as the mean ± standard deviation. Statistical analyses between treated and control samples were calculated using t-test or one-way analysis of variance (One-way ANOVA) with Tukey’s post hoc test by Graphpad Prism 6. Statistical significance was set at *p* < 0.05 or *p* < 0.01. Additionally, we performed a cluster analysis by Heatmap Illustrator. The hierarchical cluster analysis was calculated using the Pearson distances and the clustering method was the average linkage method between different toxicity assessments.

## Results and Discussion

### Bioluminescence inhibition of wastewater samples

**Figure 2:**
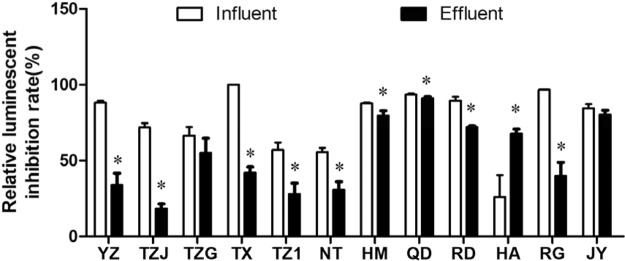
Bioluminescence inhibition rate (%) induced by the influents and effluents in the 12 WWTPs. All the data were shown as the mean ± standard deviation. The statistical analyses were calculated by using t-test. *Means the *p* < 0.05 compared to the influent.

Bioluminescence inhibition rates of influent and effluent from 12 WWTPs are shown in Fig. 2. For the influents, the highest bioluminescence inhibition was found in the TX, which was 100%, followed by the RG (96.7%) and QD (93.5%). The lowest inhibition was found in the HA (25.9%). After water treatment process, the highest and lowest bioluminescence inhibition was found in the QD (91%) and TZJ (18.5%). It should be noted that WWTPs increased bioluminescence inhibition from 25.9% to 67.8% in the HA, which uses the SBR treatment process. All the other WWTPs decreased bioluminescence inhibition rate in the range of 2.46–58.11%.

### Acute toxicity of three model organisms

**Figure 3:**
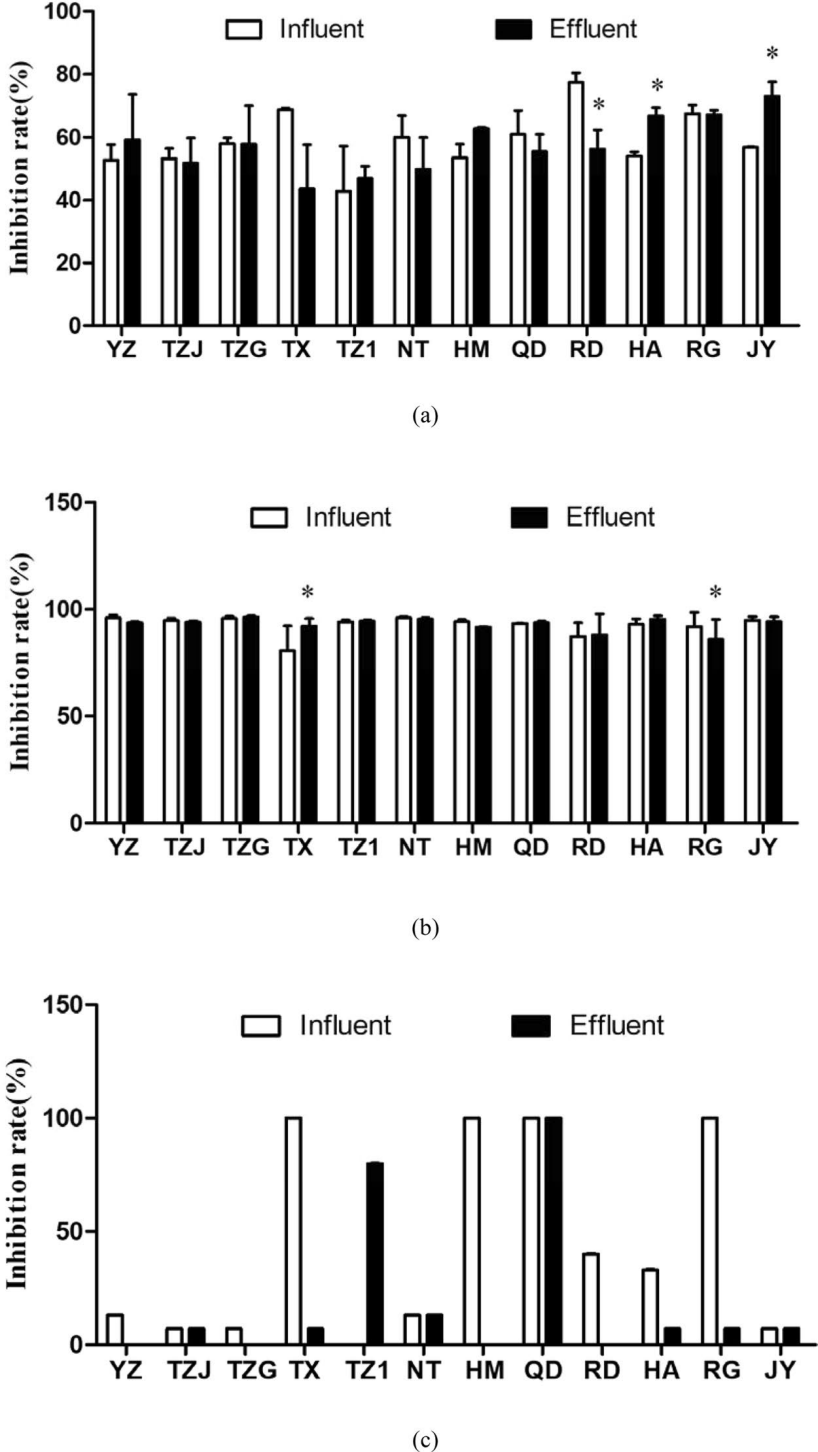
Acute toxicity induced by the influents and effluents in the 12 WWTPs. (**a**) Ggrowth inhibition rate of *E*. *gracilis* after 96-h exposure. (**b**) Growth inhibition rate of *T*. *Thermiphila* after 48- exposure. (**c**) Immobilization inhibition rate of *D*. *magna* after 48-h exposure. All the data were shown as the mean ± standard deviation. The statistical analyses were calculated by using t-test. *Means the *p* < 0.05 compared to the influent.

Microalgae *E*. *gracilis* was chosen as representation of phytoplankton. Growth inhibition rates of *E*. *gracilis* in the influents and effluents of 12 WWTPs ranged from 42.82% to 77.33% (Fig. 3a). The acute toxicity of effluent from WWTP in the RD showed a significant decrease (*p* < 0.05) compared with the influent, while WWTPs in the HA and JY increased the inhibition effect of wastewater. For the other WWTPs, no significant change in growth inhibition of *E*. *gracilis* was found. For *T*. *Thermiphila*, the growth inhibition rates in the influents and effluents were in the range of 69.73–96.00% and 75.87–95.90%, respectively (Fig. 3b). We found the WWTP in the TX increased the effect of wastewater on growth of *T*. *Thermiphila*, while the WWTP in the RG decreased the toxicity. Other WWTPs did not change the effects of wastewater.

In terms of the acute toxicity assays on *D*. *magna*, a high level of toxicity was found in the influents in the TX, HM, QD and RG with the immobilization inhibition rate reaching 100% after 48 h exposure (Fig. 3c). After treatment of WWTPs, the immobilization inhibition rate in the effluents of the TX, HM and RG were significantly decreased. However, the inhibition rate in the QD was also 100%. It should be noted that the effluent in the TZ1 had high inhibition but no obvious inhibition was found in the influent, showing a negative outcome for *D*. *magna*. From the results, we found limitations in the toxicity test on *D*. *magna* with undiluted whole effluents because most of the treatment groups are either uninhibited or totally inhibited, but this method might be suitable for roughly ranking the toxicity of wastewater samples or for rapid evaluation. Additionally, above the acute toxicity results of three types of organisms had different responses to the wastewater samples, indicating that the battery tests at different trophic levels are necessary.

### Genotoxicity of Vicia faba and *Euglena gracilis*

**Figure 4:**
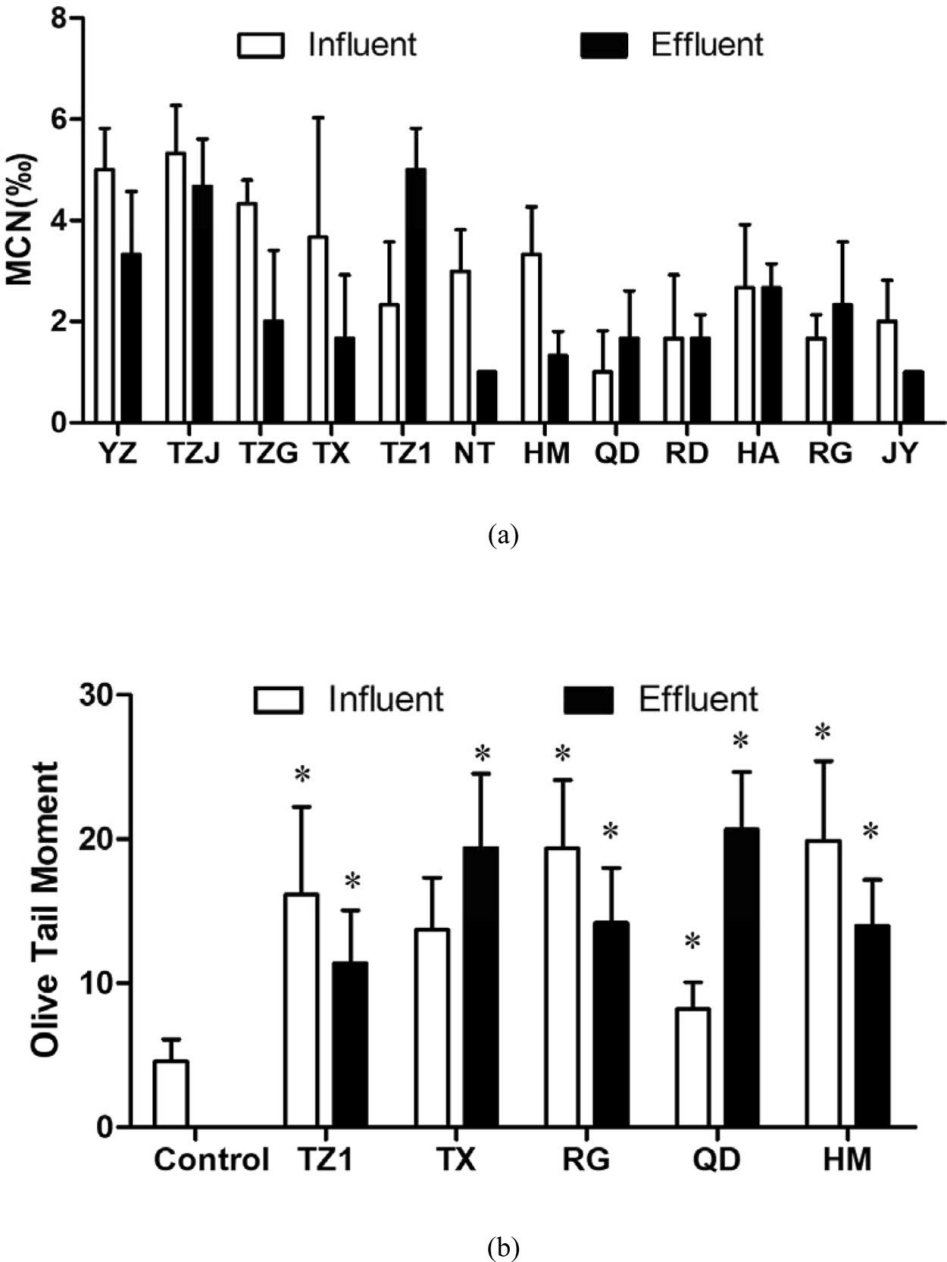
Genotoxicity induced by the influents and effluents in the 12 WWTPs. (**a**) Pollution index (MCN*‰*) of *V*. *faba* after 24-h exposure. (**b**) Olive Tail Moment of *E*. *gracilis* after 30-min exposure. All the data were shown as the mean ± standard deviation. The statistical analyses were calculated by using t-test. *Means the *p < *0.05 compared to the influent.

Results of micronucleus test on root tip cells of *V*. *faba* are showed in Fig. 4a. The PI of 24 water samples ranged from 0.38 to 2.00. Compared with the study by Liu *et al*.^27^ where the PI of effluents from two WWTPs and a river were calculated as 2.01–2.90, this study indicated a relatively low level of genetic toxicity on *V*. *faba* for most of the wastewater samples, and no significant differences were found between the influents and effluents of WWTPs.

Further, influent and effluent samples from WWTPs in the TZ1, TX, RG, QD and HM were chosen to determine genotoxicity on *E*. *gracilis*, since the influents in these WWTPs showed an increasing ratio of industrial wastewater (0, 30%, 60%, 90%, 100%, respectively) and relatively great difference in toxicity between influent and effluent (Fig. 4b). All the influents and effluents from the five WWTPs induced obvious increase of OTMs compare to control group (tap water), indicating DNA damages of *E*. *gracilis*. Wastewater treatment processes in the TZ1, RG and HM significantly decreased the OTMs induced by influents, but increased those in TX and QD.

### Cytotoxicity of WWTP effluents

**Figure 5:**
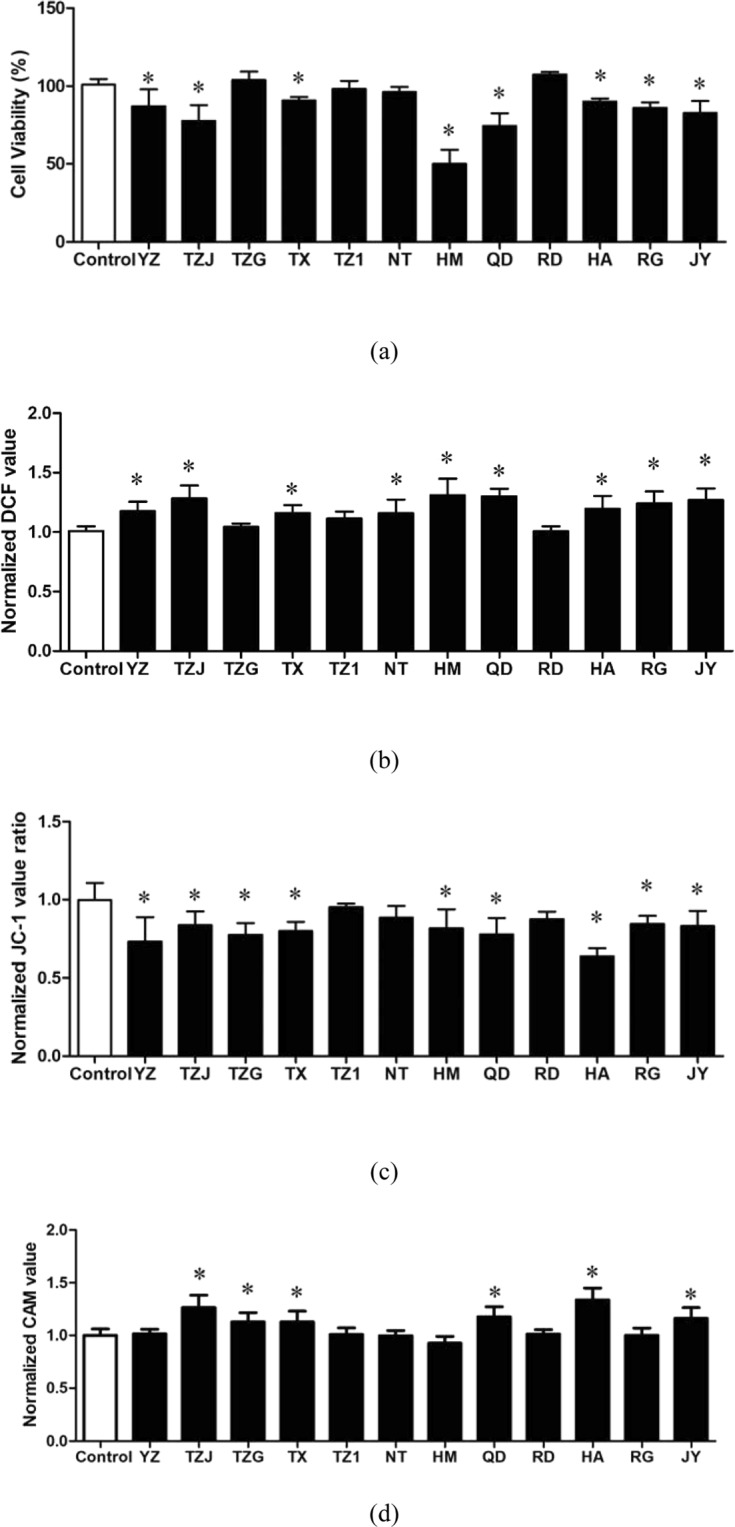
Cytotoxicity induced by the effluents in the 12 WWTPs on HepG2 cells. (**a**) Cell viability; (**b**) Generation of intracellular ROS; (**c**) Mitochondrial depolarization. (**d**) Membrane ABC transporter activity. All the data were shown as the mean ± standard deviation. The statistical analyses were calculated by using ANOVA with Tukey’s post hoc test. *Means the *p* < 0.05 compared to the control group.

Compared to above toxicity assays, human cell-based assays may be a preferable choice in the future where sensitivity is critical, such as in drinking and recycled water monitoring, which have been suggested in a previous study^28^. In this study, only the effluents were applied to perform the cytotoxicity assay based on HepG2 cells. After 24-h exposure of 12 wastewater samples at 100× concentration, eight of them significantly decreased cell viability of HepG2 (Fig. 5a). The lowest cell viability was found in the HM effluent, followed by the QD and TZJ. However, the effluents in the TZG, TZ1, NT and RD displayed no obvious cytotoxicity.

Oxidative stress response clearly stands out as a highly responsive defense mechanism, so, the intracellular ROS level were analyzed to indicate the oxidative stress induced by the effluents. Nine of 12 effluents significantly increased intracellular ROS generation compared to the control (*p* < 0.05) based on the increase of intracellular DCF fluorescence values (Fig. 5b). The high intracellular ROS levels were found in the effluent of HM, QD and TZJ, but the effluent in the TZG, TZ1 and RD did not induce obvious change. Furthermore, the mitochondrial depolarization (early indicator of cell apoptosis) was determined by decrease of red/green fluorescence intensity ratio of JC1. The results showed that the effluents of nine WWTPs significantly decreased the red/green ratio of JC1 compared to the control group (Fig. 5c), indicating the potential cell apoptosis. The effluents in the TZ1, NT and RD did not induce obvious change in mitochondrial depolarization.

Increased ROS generation has been shown to be one of the primary mechanisms by which pollutants induce cytotoxicity^29^. Moreover, ROS generation is linked to mitochondrial oxidative damage and apoptosis. Based on the above results of this study, cell viability usually changed similarly with the ROS generation and mitochondrial depolarization induced by all the samples. For example, all the three toxic endpoints indicated that the effluents in the HM, QD and TZJ could induce high toxicity, while the effluent of the TZG, TZ1, NT and RD induced low toxicity. Thus, it can be deduced that wastewater samples increased ROS generation and damaged cell function (mitochondrial membrane potential) to induce cytotoxicity (decrease of cell viability) when exposed to HepG2 cells.

### Inhibition of ABC transporter activity induced by WWTP effluents

Intracellular accumulation of fluorescence value of CAM in cells was applied to indicate the inhibition of ABC transporter activity. Among the effluents of 12 WWTPs, six effluent samples significantly inhibited ABC transporter activity, including the TZJ, TZG, TX, QD, HA and JY (Fig. 5d). Furthermore, we found that the influents in the TZJ, TX, HA and JY mainly consisted of wastewater from machine manufacturing and new material industries. Therefore, the products of these industries might play important roles in the inhibition of ABC transporter activity, which need to be further studied.

Inhibition of ABC transporter could induce accumulation of its substrate and act as chemosensitizer^30^. In this study, we chose As as target pollutant to identify the chemosensitive effect, since As within the cell can be conjugated by GSH into As-GSH, which is a substrate of ABCCs/MRPs^31^. Only six wastewater samples inhibiting ABC transporter activity significantly increased generation of intracellular ROS level induced by As at nontoxic concentration (Fig. S1). These results showed that the effluents that inhibited ABC transporter activity could induce chemosensitive effect, which need to be considered during risk assessment of the effluents.

### Correlation analyses of toxicity at different endpoints

A battery of bioassays include integrative endpoints which are potentially more effective in evaluating water quality and process efficiency, given that these are highly variable and complex mixtures^32^. In this study, nine toxic endpoints involving in microorganism, phytoplankton, zooplankton, plant and human were applied. Correlation coefficient was calculated to determine whether the different toxicity tests could be replaced by one another. Results shown in Table S4 indicated that very low correlation coefficient was obtained among toxicity data, except the correlation between cell viability and intracellular ROS level. These results might be due to the different response in different species, indicating that battery of toxicity assays is very necessary to completely characterize the toxicity and risk of wastewater.

**Figure 6:**
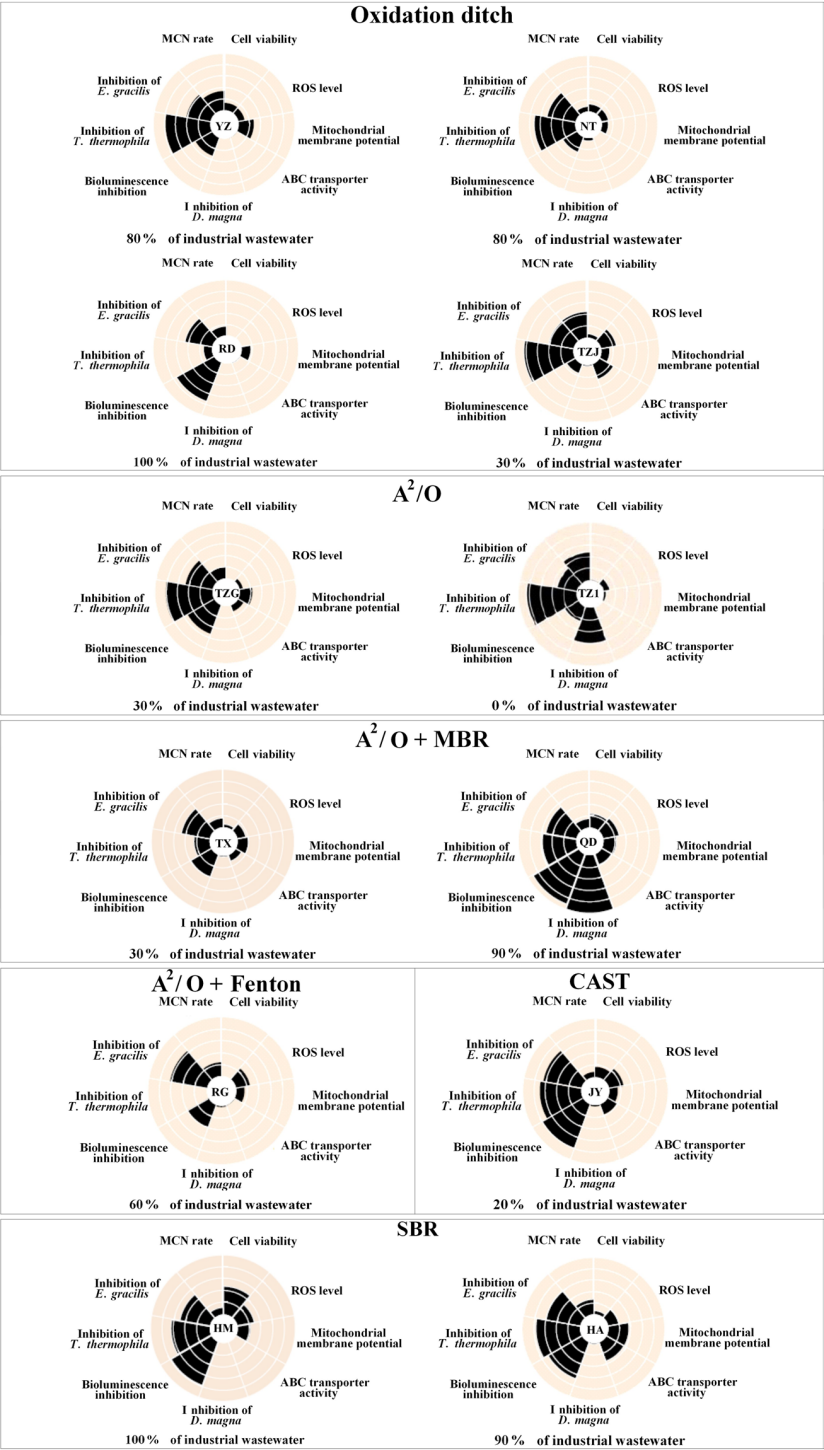
Normalized toxicity data in the effluents of 12 WWTPs. A total of 9 toxic endpoints involving microorganism, phytoplankton, zooplankton, plant and human cell lines were applied. The toxicity data of each tests in water samples was normalized based on that in the control group. The figures were classified based on the treatment technologies.

The toxicity data of each tests in water samples was further normalized based on that in the control group. Then, these normalized data were combined and shown in Fig. 6. Based on the figure, we could found that no correlation between the effluent toxicity and the ratio of industrial to domestic wastewater was found. However, the treatment technologies had higher influence on effluent toxicity. The effluent from SBR and cyclic activated sludge system (CAST) treatment processes always had higher toxicity than other treatment process. The lowest normalized toxicity data was found in the WWTPs where A^2^/O + membrane bioreactor (MBR) and A^2^/O + Fenton treatment processes were used. The only exception was the QD that used the A^2^/O + MBR process. The good removal efficiency of A^2^/O -based process could also be reported in the literatures^33,34^. For example, Shi *et al*. found that the A^2^/O -based process was efficient in acute toxicity removal of coking wastewater^35^. The A^2^/O -based processes might be good choice to reduce the toxicity of industrial wastewater. However, the conclusions should be further confirmed based on the more A^2^/O –based WWTPs. The under mechanisms should be also analyzed. The effect-direct analysis (EDA) that combines the effect testing, fractionation and chemical analysis might be promising tool for identifying predominant pollutants in wastewater and characterize the reason of high toxicity reduction of A^2^/O –based process. On the other hand, our current results and literatures can indicate that the SBR and CAST might be not good to effectively reduce the toxicity of industrial wastewater^36,37^. The low toxicity reduction might be due to that the SBR and CAST-processes are the combination of the procedural functions “aeration” and “clarification” in one reaction tank, which might reduce the detoxification of chemicals by microorganisms, resulting in higher toxicity of effluent.

**Figure 7:**
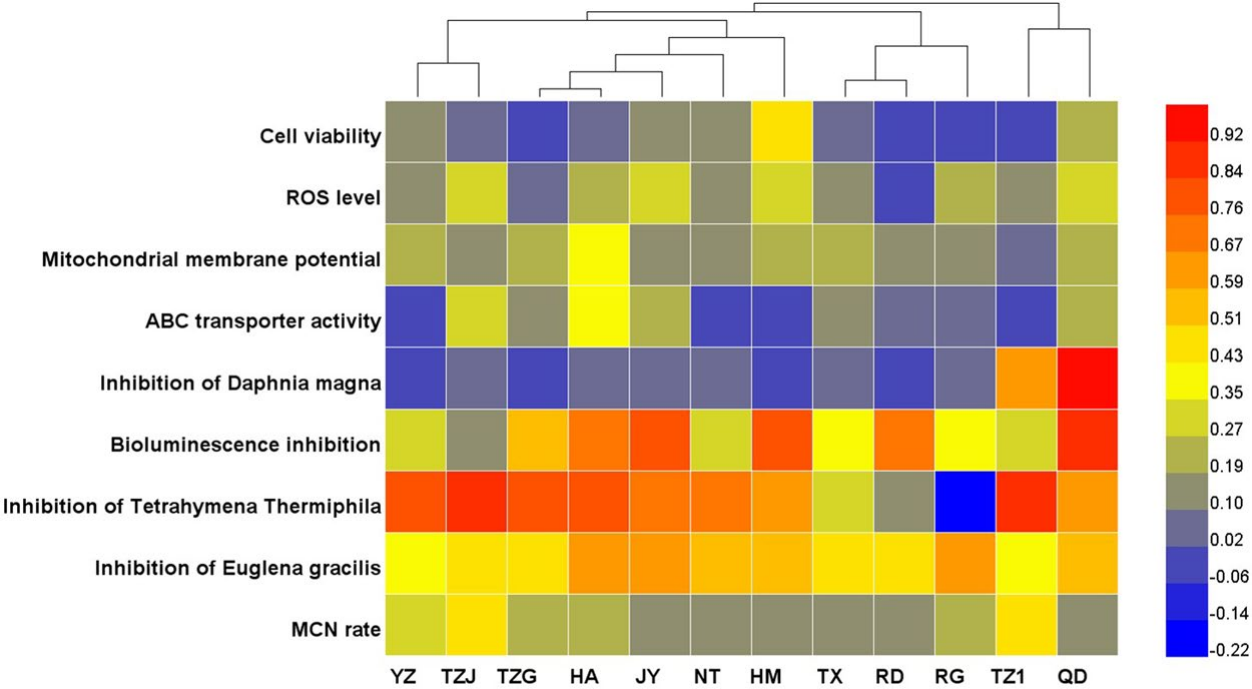
Cluster analyses of toxicity data on different species for the 12 WWTPs. The cluster analysis was performed by Heatmap Illustrator.

In order to identify above conclusions, we further analyzed the toxicity data by heatmap analysis. As shown in the Fig. 7, the 12 WWTPs could be clustered four groups. The first two groups including 7 WWTPs (YZ, TZJ, TZG, HA, JY, NT and HM) mainly applied the oxidation ditch and SBR-process. For example, YZ and TZJ were clustered together, because both of them involved the oxidation ditch-process. Thus, the similarity between them may originate from the same wastewater treatment process. Aother two groups (TX, RG, TZ1 and QD) mainly used the A^2^/O-process. Above results further showed that the treatment process might play important roles in the water quality and effluent toxicity.

## Conclusions

All influents from 12 industrial WWTPs induced high toxicity. Most of the wastewater treatment processes significantly decreased the toxicity of influents. However, the effluents in HA, JY, TX, TZ1 and QD had higher toxicity in one or more toxic endpoints compared to the influents. More importantly, whatever the difference components in the influents, the effluents from the same type of treatment process had high similarity, and SBR-based processes might be not good to reduce toxicity of industrial wastewater. Additionally, this study also found some effluents could inhibit membrane ABC transporter activity and induce chemosenstive effects. Results of this study provide new understanding to the relationship among battery toxicities and treatment process of industrial park WWTPs, which is very useful for the design, management and operation of industrial park WWTPs.

## Supplementary information

Supporting information
