# Supplementary material for: Comparative analysis of toxicity reduction of wastewater in twelve industrial park wastewater treatment plants based on battery of toxicity assays
Source: Sci Rep. 2019 Mar 6;9:3751. doi: 10.1038/s41598-019-40154-z (PMC6403317; doi:10.1038/s41598-019-40154-z)
Supplement: Supplementary file 1 — Supporting information [file 41598_2019_40154_MOESM1_ESM.docx]

**Supplementary material for**

**Comparative analysis of toxicity reduction of wastewater in twelve industrial park wastewater treatment plants based on battery of toxicity assays**

Yue Yu, Bing Wu*, Linmiao Jiang, Xu-Xiang Zhang, Hong-Qiang Ren, Mei Li*

State Key Laboratory of Pollution Control and Resource Reuse, School of the Environment, Nanjing University, Nanjing, 210023, P.R. China

* Corresponding author:

E-mail: bwu@nju.edu.cn (B. Wu); [meili@nju.edu.cn](mailto:meili@nju.edu.cn) (M. Li)

Postal address: NO. 163 Xianlin Avenue, Nanjing, 210023, P.R. China

**Table S1** Basic information on the WWTPs in 12 industrial parks

| WWTPs | Primary treatment ^a^ | Secondary Treatment ^b^ | Tertiary treatment ^c^ | % of industrial wastewater | Industrial type |
| --- | --- | --- | --- | --- | --- |
| YZ | GC | OD | - | 80% | Pesticide, chemical, material, petroleum |
| TZJ | GC | OD | - | 20%-30% | Petroleum, pharmaceuticals, material, mechatronics, chemical |
| TZG | GC | A^2^/O | - | 20%-30% | Grain & oil |
| TX | GC | A^2^/O +MBR | O_3_ | 30% | Printing&dyeing, mechatronics, chemical |
| TZ1 | GC | A^2^/O | ClO_2_ | 0% |  |
| NT | GC | OD | - | 70-80% | Mechatronics, material, pharmaceuticals, energy |
| HM | GC | SBR | - | 100% | Printing&dyeing, fermentation, pharmaceutical |
| QD | GC | A^2^/O + MBR | - | 90% | Chemical, pesticide, pharmaceuticals |
| RD | GC | OD | - | 100% | Chemical, pesticide |
| HA | GC | SBR | - | 90% | Chemical, energy |
| RG | GC | A^2^/O | Fenton | 60% | Pharmaceutical, pesticide, material, petroleum, chemical |
| JY | GC | CAST | - | 20% | Printing&dyeing, mechanical, chemical |

^a^ GC: grit chamber

^b^ OD: oxidation ditch; A^2^/O: anaerobic/anoxic/oxic; MBR: Membrane bioreactor; SBR: Sequencing batch reactor; CAST: Cyclic activated sludge system

^c^ – means no tertiary treatment or the tertiary treatment was not in operation when the samples were collected. ClO_2_: ClO_2_ disinfection; O_3_: ozonation

**Table S2** The pH values and alkalinity in the influents of 12 WWTPs

|  |  | YZ | TZJ | TZG | TX | TZ1 | NT | HM | QD | RD | HA | RG | JY |
| --- | --- | --- | --- | --- | --- | --- | --- | --- | --- | --- | --- | --- | --- |
| pH value | Influent | 7.81 | 7.99 | 7.43 | 7.65 | 7.52 | 7.99 | 6.34 | 6.63 | 7.72 | 8.45 | 7.84 | 6.73 |
|  | Effluent | 7.84 | 8.08 | 7.61 | 7.72 | 7.48 | 7.74 | 6.25 | 6.75 | 8.05 | 8.21 | 8.43 | 6.92 |
| Alkalinity  (CaO, mg/L) | Influent | 125.06 | 187.59 | 162.58 | 275.13 | 137.56 | 125.06 | 262.62 | 331.40 | 262.62 | 175.08 | 375.18 | 293.89 |
|  | Effluent | 131.31 | 81.29 | 175.08 | 243.86 | 100.05 | 137.56 | 644.05 | 300.14 | 256.37 | 212.60 | 81.29 | 156.32 |

**Table S3** Concentrations (mg/L) of chemical oxygen demand (COD), total nitrogen (TN) and total phosphorus (TP) in the effluents of 12 WWTPs chemical analyses of water samples. All the data was shown as mean from three independent tests.

|  | YZ | TZJ | TZG | TX | TZ1 | NT | HM | QD | RD | HA | RG | JY |
| --- | --- | --- | --- | --- | --- | --- | --- | --- | --- | --- | --- | --- |
| COD | 8.84±2.77 | 28.65±3.77 | 16.46±2.94 | 85.33±4.35 | 14.99±3.77 | 83.56±14.00 | 87.78±18.13 | 80.00±11.52 | 60.44±10.96 | 83.56±10.06 | 80.00±7.54 | 62.22±10.06 |
| TN | 9.14±0.07 | 18.66±0.36 | 9.06±0.13 | 6.63±0.12 | 5.84±0.14 | 14.48±0.34 | 25.62±0.39 | 12.83±0.23 | 16.65±0.13 | 3.48±0.09 | 15.25±0.33 | 8.06±0.07 |
| TP | 0.15±0.01 | 0.09±0.01 | 0.63±0.03 | 0.21±0.01 | 0.06±0.01 | 0.42±0.09 | 0.51±0.11 | 0.37±0.02 | 0.16±0.01 | 0.11±0.01 | 0.03±0.00 | 0.13±0.01 |

**Table S4** Correlation coefficient among toxicities data as determined in this study.

|  | Cell viability | ROS level | Mitochondrial membrane potential | ABC transporter activity | Daphnia |
| --- | --- | --- | --- | --- | --- |
| Cell viability | 1 | 0.680* | 0.215 | -0.150 | 0.160 |
| ROS level |  | 1 | 0.139 | 0.326 | 0.231 |
| Mitochondrial membrane potential |  |  | 1 | 0.476 | -0.147 |
| ABC transporter activity |  |  |  | 1 | 0.093 |
| Photobacterium | 0.459 | 0.258 | 0.435 | 0.153 | 0.245 |
| Tetrahymena | 0.252 | 0.051 | 0.120 | 0.291 | 0.153 |
| Algae | 0.263 | 0.521 | 0.362 | 0.312 | -0.143 |
| MCN | -0.351 | -0.071 | -0.231 | 0.171 | 0.177 |

* means *p*<0.05.


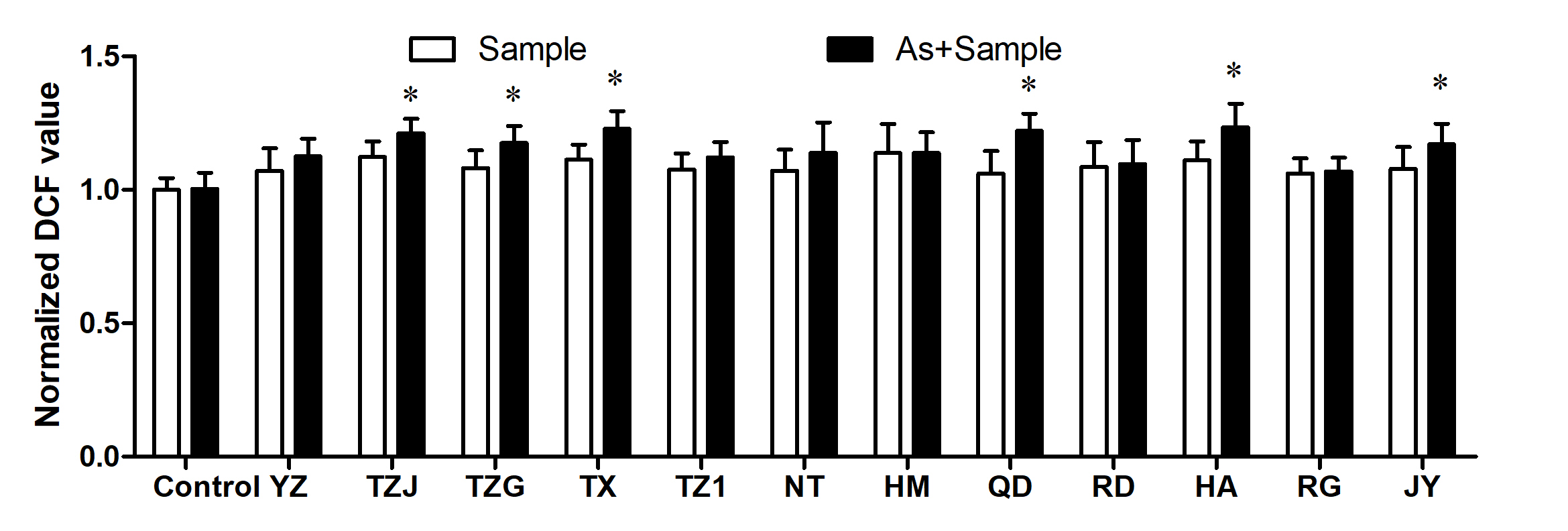


**Figure S1** Influence of wastewater samples on arsenic toxicity. All the data were shown as the mean ± standard deviation. The statistical analyses were calculated using t-test. * means the *p* < 0.05 compared to the influent.
